# *N*-[4-Chloro-2-methyl-6-(*N*-methyl­car­bam­oyl)phen­yl]-1-(3-chloro-2-pyrid­yl)-3-trifluoro­meth­yl-1*H*-pyrazole-5-carboxamide

**DOI:** 10.1107/S1600536808037604

**Published:** 2008-11-20

**Authors:** Huibin Yang, Haibo Yu, Bin Li

**Affiliations:** a Agrochemicals Division, Shenyang Research Institute of Chemical Industry, Shenyang 110021, People’s Republic of China

## Abstract

In the title compound, C_19_H_14_Cl_2_F_3_N_5_O_2_, which shows insecticidal activity, the dihedral angle between the pyrazole and pyridine rings is 68.15 (16)°. In the crystal structure, the mol­ecules are linked by N—H⋯O and C—H⋯O hydrogen bonds and an intra­molecular N—H⋯O inter­action also occurs. The F atoms of the –CF_3_ group are disordered over two sets of sites in a 0.800 (8):0.200 (8) ratio.

## Related literature

For the synthesis and background to the insecticidal properties of the title compound, see: Lahm *et al.* (2003 ▶, 2005 ▶).
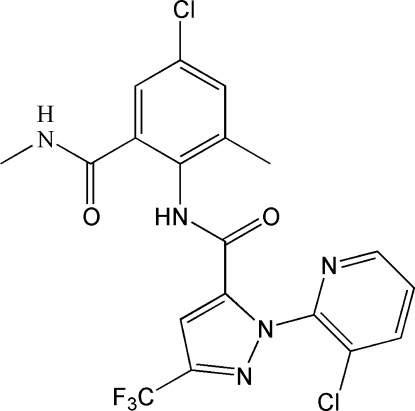


## Experimental

### 

#### Crystal data

C_19_H_14_Cl_2_F_3_N_5_O_2_
*M* *_r_* = 472.25
Orthorhombic, 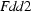

*a* = 26.4612 (16) Å
*b* = 32.8657 (19) Å
*c* = 9.4679 (6) Å
*V* = 8233.9 (9) Å^3^
*Z* = 16
Mo *K*α radiation
μ = 0.37 mm^−1^
*T* = 296 (2) K
0.24 × 0.20 × 0.18 mm

#### Data collection

Bruker SMART CCD diffractometer
Absorption correction: multi-scan (*SADABS*; Bruker, 1999 ▶) *T*_min_ = 0.917, *T*_max_ = 0.937
10439 measured reflections
3565 independent reflections
3284 reflections with *I* > 2σ(*I*)
*R*_int_ = 0.021

#### Refinement

*R*[*F* ^2^ > 2σ(*F* ^2^)] = 0.034
*wR*(*F* ^2^) = 0.082
*S* = 1.04
3565 reflections
310 parameters
49 restraints
H-atom parameters constrained
Δρ_max_ = 0.18 e Å^−3^
Δρ_min_ = −0.20 e Å^−3^
Absolute structure: Flack (1983 ▶), 1621 Friedel pairs
Flack parameter: −0.01 (7)

### 

Data collection: *SMART* (Bruker, 1999 ▶); cell refinement: *SAINT* (Bruker, 1999 ▶); data reduction: *SAINT*; program(s) used to solve structure: *SHELXS97* (Sheldrick, 2008 ▶); program(s) used to refine structure: *SHELXL97* (Sheldrick, 2008 ▶); molecular graphics: *SHELXTL* (Sheldrick, 2008 ▶); software used to prepare material for publication: *SHELXTL*.

## Supplementary Material

Crystal structure: contains datablocks I, global. DOI: 10.1107/S1600536808037604/hb2777sup1.cif

Structure factors: contains datablocks I. DOI: 10.1107/S1600536808037604/hb2777Isup2.hkl

Additional supplementary materials:  crystallographic information; 3D view; checkCIF report

## Figures and Tables

**Table 1:** Hydrogen-bond geometry (Å, °)

*D*—H⋯*A*	*D*—H	H⋯*A*	*D*⋯*A*	*D*—H⋯*A*
N4—H4⋯O2	0.86	2.37	2.673 (3)	101
N4—H4⋯O2^i^	0.86	2.25	3.107 (3)	176
N5—H5⋯O1^ii^	0.86	2.16	2.915 (3)	146
C8—H8⋯O2^i^	0.93	2.50	3.168 (4)	129

Symmetry codes: (i) 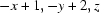; (ii) 
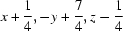
.

## supplementary crystallographic information

### Comment

Anthranilamide compounds containing an N-pyridyl pyrazole grouping are a new class of inseticides showing potent activity against a broad spectrum of Lepidoptera and no-cross resistance to existing insecticides (Lahm *et al.*,  2003) and their mode of action has been been established (Lahm *et al.*,  2005). Anthranilamides show little effect on mammalian ryanodine receptors and as a result they show excellent insect *versus* mammalian selectivity. As part of our studies in this area, we now report the crystal structures of the title compound, (I), (Fig. 1) which possesses high insecticidal activity.

The pyrazole ring and pyridine rings are not coplanar, the dihedral angle formed by the least-squares planes of the rings being equal to 68.15 (16)°.  The dihedral angle between the mean plane of the pyrazole ring and the plane of the C10/O1/N4 group is 23.4 (2)° and the dihedral angle between the mean plane of the phenyl ring and the plane of the C10/O1/N4 group is 59.9 (2)°.

An intramolecular N—H···O hydrogen bond (Table 1) is observed, which helps to establish the molecular conformation.  Intermolecular N—H···O and C—H···O bonds result in a three-dimensional network.

### Experimental

The title compound was prepared by the literature method (Lahm *et al.*, 2003). The crude products were purified by silica-gel column chromatography and then grown from acetone to afford colourless blocks of (I).

Anal. Calcd for C_12_H_14_N_4_O_3_: C, 48.32; H, 2.99; N, 14.83. Found: C, 48.47; H, 3.04; N, 14.66. ^1^H NMR(CDCl_3_): 2.19 (s, 3H, CH_3_), 2.70 (d, J=4.5 Hz, 3H, CH_3_), 7.30–7.31 (m, 2H, Ph), 7.57 (dd, 1H), 8.05 (d, J=8.1 Hz, 1H), 8.22 (br s, J=4.5 Hz, 1H, NH), 8.46 (d, J=4.8 Hz, 1H), 7.64 (s, 1H), 10.43 (s, 1H, NH).

### Refinement

Although all H atoms were visible in difference maps, they were placed in geometrically calculated positions, with C—H distances in the range 0.93–0.96Å and N—H distances of 0.86 Å, andincluded in the final refinement in the riding model approximation,with *U*_iso_(H) = 1.2*U*_eq_(C, N) or 1.5*U*_eq_(methyl C).  The F atoms of the -CF_3_ are disordered over two sets of sites in a 0.800 (8):0.200 (8) ratio.

### Crystal data

**Table:**

C_19_H_14_Cl_2_F_3_N_5_O_2_	*D*_x_ = 1.524 Mg m^−^^3^
*M**_r_* = 472.25	Melting point: 492(1) K
Orthorhombic, *F**d**d*2	Mo *K*α radiation, λ = 0.71073 Å
*a* = 26.4612 (16) Å	Cell parameters from 3952 reflections
*b* = 32.8657 (19) Å	θ = 2.4–23.9°
*c* = 9.4679 (6) Å	µ = 0.37 mm^−^^1^
*V* = 8233.9 (9) Å^3^	*T* = 296 K
*Z* = 16	Block, colourless
*F*(000) = 3840	0.24 × 0.20 × 0.18 mm

### Data collection

**Table:**

Bruker SMART CCD diffractometer	3565 independent reflections
Radiation source: fine-focus sealed tube	3284 reflections with *I* > 2σ(*I*)
graphite	*R*_int_ = 0.021
ω scans	θ_max_ = 25.0°, θ_min_ = 2.0°
Absorption correction: multi-scan (*SADABS*; Bruker, 1999)	*h* = −31→24
*T*_min_ = 0.917, *T*_max_ = 0.937	*k* = −34→38
10439 measured reflections	*l* = −11→11

### Refinement

**Table:**

Refinement on *F*^2^	Secondary atom site location: difference Fourier map
Least-squares matrix: full	Hydrogen site location: inferred from neighbouring sites
*R*[*F*^2^ > 2σ(*F*^2^)] = 0.034	H-atom parameters constrained
*wR*(*F*^2^) = 0.082	*w* = 1/[σ^2^(*F*_o_^2^) + (0.0387*P*)^2^ + 8.8659*P*] where *P* = (*F*_o_^2^ + 2*F*_c_^2^)/3
*S* = 1.04	(Δ/σ)_max_ = 0.001
3565 reflections	Δρ_max_ = 0.18 e Å^−^^3^
310 parameters	Δρ_min_ = −0.20 e Å^−^^3^
49 restraints	Absolute structure: Flack (1983), 1621 Friedel pairs
Primary atom site location: structure-invariant direct methods	Flack parameter: −0.01 (7)

### Special details

**Table:**

Geometry. All e.s.d.'s (except the e.s.d. in the dihedral angle between two l.s. planes) are estimated using the full covariance matrix. The cell e.s.d.'s are taken into account individually in the estimation of e.s.d.'s in distances, angles and torsion angles; correlations between e.s.d.'s in cell parameters are only used when they are defined by crystal symmetry. An approximate (isotropic) treatment of cell e.s.d.'s is used for estimating e.s.d.'s involving l.s. planes.
Refinement. Refinement of *F*^2^ against ALL reflections. The weighted *R*-factor *wR* and goodness of fit *S* are based on *F*^2^, conventional *R*-factors *R* are based on *F*, with *F* set to zero for negative *F*^2^. The threshold expression of *F*^2^ > σ(*F*^2^) is used only for calculating *R*-factors(gt) *etc*. and is not relevant to the choice of reflections for refinement. *R*-factors based on *F*^2^ are statistically about twice as large as those based on *F*, and *R*- factors based on ALL data will be even larger.

### Fractional atomic coordinates and isotropic or equivalent isotropic displacement parameters (Å^2^)

**Table:**

	*x*	*y*	*z*	*U*_iso_*/*U*_eq_	Occ. (<1)
Cl1	0.28309 (3)	1.00598 (3)	0.40561 (10)	0.0647 (3)	
Cl2	0.46782 (4)	0.85540 (5)	−0.17854 (12)	0.1075 (5)	
F1	0.39895 (14)	1.06805 (12)	0.8595 (6)	0.1059 (17)	0.800 (8)
F2	0.4312 (2)	1.01859 (14)	0.9614 (4)	0.1148 (18)	0.800 (8)
F3	0.47071 (12)	1.04708 (16)	0.7981 (5)	0.0958 (15)	0.800 (8)
F1'	0.4028 (5)	1.0381 (5)	0.9535 (11)	0.074 (4)	0.200 (8)
F2'	0.4714 (4)	1.0263 (5)	0.8653 (18)	0.087 (5)	0.200 (8)
F3'	0.4283 (7)	1.0724 (3)	0.7878 (16)	0.106 (6)	0.200 (8)
O1	0.36097 (7)	0.91496 (6)	0.4022 (2)	0.0453 (5)	
O2	0.52136 (8)	0.96046 (6)	0.3535 (3)	0.0576 (6)	
N1	0.35642 (9)	0.99358 (8)	0.7518 (3)	0.0445 (6)	
N2	0.34753 (8)	0.97021 (6)	0.6376 (2)	0.0360 (5)	
N3	0.28654 (9)	0.92344 (8)	0.7109 (3)	0.0479 (6)	
N4	0.42127 (8)	0.95772 (6)	0.3170 (2)	0.0342 (5)	
H4	0.4359	0.9808	0.3291	0.041*	
N5	0.56488 (8)	0.90701 (7)	0.2779 (3)	0.0419 (6)	
H5	0.5672	0.8873	0.2186	0.050*	
C1	0.26575 (11)	0.96695 (9)	0.5174 (3)	0.0448 (7)	
C2	0.21856 (11)	0.94974 (11)	0.5056 (4)	0.0566 (9)	
H2	0.1958	0.9587	0.4375	0.068*	
C3	0.20605 (12)	0.91908 (11)	0.5966 (4)	0.0602 (10)	
H3	0.1747	0.9064	0.5899	0.072*	
C4	0.23980 (14)	0.90720 (10)	0.6975 (4)	0.0592 (9)	
H4A	0.2302	0.8869	0.7604	0.071*	
C5	0.29811 (10)	0.95245 (8)	0.6209 (3)	0.0379 (6)	
C6	0.40279 (10)	1.00776 (9)	0.7321 (3)	0.0434 (7)	
C7	0.42585 (12)	1.03518 (11)	0.8368 (4)	0.0596 (9)	
C8	0.42392 (10)	0.99401 (9)	0.6056 (3)	0.0429 (7)	
H8	0.4558	1.0000	0.5695	0.052*	
C9	0.38752 (10)	0.96987 (8)	0.5464 (3)	0.0346 (6)	
C10	0.38824 (9)	0.94496 (7)	0.4152 (3)	0.0329 (6)	
C11	0.43316 (10)	0.93476 (7)	0.1941 (3)	0.0323 (6)	
C12	0.48281 (9)	0.92011 (7)	0.1738 (3)	0.0319 (6)	
C13	0.49293 (10)	0.89622 (9)	0.0565 (3)	0.0415 (6)	
H13	0.5254	0.8864	0.0410	0.050*	
C14	0.45484 (12)	0.88718 (10)	−0.0367 (3)	0.0534 (8)	
C15	0.40693 (11)	0.90324 (10)	−0.0207 (3)	0.0507 (8)	
H15	0.3822	0.8976	−0.0875	0.061*	
C16	0.39557 (10)	0.92751 (9)	0.0935 (3)	0.0401 (6)	
C17	0.34412 (11)	0.94686 (11)	0.1030 (4)	0.0565 (8)	
H17A	0.3290	0.9476	0.0108	0.085*	
H17B	0.3474	0.9741	0.1385	0.085*	
H17C	0.3232	0.9313	0.1656	0.085*	
C18	0.52432 (10)	0.93058 (8)	0.2762 (3)	0.0334 (6)	
C19	0.60538 (12)	0.91391 (11)	0.3778 (4)	0.0597 (9)	
H19A	0.6216	0.9394	0.3571	0.090*	
H19B	0.6296	0.8923	0.3708	0.090*	
H19C	0.5918	0.9147	0.4718	0.090*	

### Atomic displacement parameters (Å^2^)

**Table:**

	*U*^11^	*U*^22^	*U*^33^	*U*^12^	*U*^13^	*U*^23^
Cl1	0.0572 (5)	0.0631 (5)	0.0737 (6)	0.0044 (4)	−0.0053 (4)	0.0203 (5)
Cl2	0.0781 (7)	0.1621 (12)	0.0821 (8)	0.0360 (7)	−0.0273 (6)	−0.0839 (8)
F1	0.090 (2)	0.090 (2)	0.137 (3)	0.0050 (19)	−0.020 (2)	−0.063 (2)
F2	0.168 (4)	0.117 (3)	0.059 (2)	−0.042 (3)	−0.040 (2)	−0.0052 (18)
F3	0.067 (2)	0.132 (3)	0.088 (3)	−0.0499 (19)	0.0139 (17)	−0.043 (2)
F1'	0.067 (6)	0.095 (6)	0.060 (6)	−0.014 (5)	0.009 (4)	−0.021 (5)
F2'	0.072 (6)	0.094 (6)	0.094 (7)	0.004 (5)	−0.017 (5)	−0.018 (5)
F3'	0.121 (8)	0.094 (7)	0.103 (7)	−0.008 (5)	−0.014 (5)	−0.004 (5)
O1	0.0485 (11)	0.0440 (11)	0.0433 (11)	−0.0146 (9)	0.0108 (10)	−0.0005 (9)
O2	0.0434 (12)	0.0537 (12)	0.0757 (17)	0.0105 (10)	−0.0185 (11)	−0.0324 (12)
N1	0.0407 (13)	0.0564 (15)	0.0365 (13)	−0.0054 (11)	0.0038 (10)	−0.0095 (11)
N2	0.0312 (11)	0.0441 (12)	0.0326 (12)	−0.0019 (9)	0.0043 (9)	−0.0051 (10)
N3	0.0511 (15)	0.0500 (14)	0.0426 (14)	−0.0052 (12)	0.0153 (11)	−0.0014 (12)
N4	0.0332 (11)	0.0331 (11)	0.0364 (13)	−0.0044 (9)	0.0052 (10)	−0.0030 (10)
N5	0.0347 (12)	0.0433 (13)	0.0478 (14)	0.0065 (10)	−0.0059 (10)	−0.0128 (11)
C1	0.0378 (16)	0.0472 (16)	0.0492 (18)	0.0027 (13)	0.0053 (13)	−0.0069 (14)
C2	0.0356 (17)	0.067 (2)	0.067 (2)	−0.0003 (15)	−0.0020 (16)	−0.0149 (18)
C3	0.0381 (17)	0.068 (2)	0.075 (2)	−0.0137 (16)	0.0124 (17)	−0.022 (2)
C4	0.065 (2)	0.0553 (19)	0.058 (2)	−0.0176 (17)	0.0271 (17)	−0.0078 (16)
C5	0.0335 (14)	0.0422 (15)	0.0380 (16)	−0.0023 (11)	0.0100 (12)	−0.0074 (12)
C6	0.0380 (16)	0.0545 (17)	0.0376 (16)	−0.0054 (13)	−0.0001 (12)	−0.0042 (13)
C7	0.0503 (19)	0.077 (2)	0.052 (2)	−0.0108 (18)	0.0062 (16)	−0.0155 (18)
C8	0.0326 (14)	0.0557 (17)	0.0405 (16)	−0.0058 (13)	0.0051 (12)	−0.0010 (13)
C9	0.0337 (14)	0.0392 (14)	0.0307 (13)	0.0022 (11)	0.0021 (11)	0.0020 (11)
C10	0.0288 (13)	0.0351 (13)	0.0347 (14)	0.0006 (11)	0.0028 (11)	0.0050 (11)
C11	0.0330 (13)	0.0329 (13)	0.0310 (14)	−0.0043 (11)	0.0016 (11)	0.0042 (10)
C12	0.0314 (13)	0.0310 (12)	0.0333 (14)	−0.0044 (10)	0.0008 (11)	0.0008 (11)
C13	0.0357 (14)	0.0499 (16)	0.0391 (15)	0.0027 (12)	0.0002 (12)	−0.0068 (13)
C14	0.0502 (18)	0.069 (2)	0.0405 (17)	0.0035 (16)	−0.0066 (13)	−0.0218 (16)
C15	0.0422 (17)	0.071 (2)	0.0392 (17)	−0.0054 (15)	−0.0108 (13)	−0.0092 (16)
C16	0.0319 (14)	0.0501 (16)	0.0383 (16)	−0.0040 (12)	0.0012 (11)	0.0034 (13)
C17	0.0365 (15)	0.085 (2)	0.0481 (19)	0.0068 (16)	−0.0051 (14)	0.0040 (16)
C18	0.0335 (14)	0.0308 (13)	0.0359 (15)	−0.0015 (11)	−0.0003 (11)	−0.0020 (11)
C19	0.0393 (17)	0.073 (2)	0.067 (2)	0.0107 (15)	−0.0141 (15)	−0.0186 (19)

### Geometric parameters (Å, °)

**Table:**

Cl1—C1	1.725 (3)	C2—H2	0.9300
Cl2—C14	1.736 (3)	C3—C4	1.365 (5)
F1—C7	1.311 (4)	C3—H3	0.9300
F2—C7	1.307 (4)	C4—H4A	0.9300
F3—C7	1.303 (4)	C6—C8	1.397 (4)
F1'—C7	1.266 (8)	C6—C7	1.472 (4)
F2'—C7	1.269 (8)	C8—C9	1.368 (4)
F3'—C7	1.310 (8)	C8—H8	0.9300
O1—C10	1.228 (3)	C9—C10	1.488 (4)
O2—C18	1.227 (3)	C11—C16	1.397 (4)
N1—C6	1.326 (4)	C11—C12	1.412 (4)
N1—N2	1.347 (3)	C12—C13	1.387 (4)
N2—C9	1.366 (3)	C12—C18	1.505 (3)
N2—C5	1.441 (3)	C13—C14	1.372 (4)
N3—C5	1.315 (4)	C13—H13	0.9300
N3—C4	1.353 (4)	C14—C15	1.382 (4)
N4—C10	1.343 (3)	C15—C16	1.377 (4)
N4—C11	1.423 (3)	C15—H15	0.9300
N4—H4	0.8600	C16—C17	1.505 (4)
N5—C18	1.324 (3)	C17—H17A	0.9600
N5—C19	1.447 (4)	C17—H17B	0.9600
N5—H5	0.8600	C17—H17C	0.9600
C1—C2	1.375 (4)	C19—H19A	0.9600
C1—C5	1.386 (4)	C19—H19B	0.9600
C2—C3	1.367 (5)	C19—H19C	0.9600
			
C6—N1—N2	104.4 (2)	F3—C7—C6	111.8 (3)
N1—N2—C9	112.1 (2)	F2—C7—C6	113.4 (3)
N1—N2—C5	118.5 (2)	F3'—C7—C6	110.7 (7)
C9—N2—C5	129.1 (2)	F1—C7—C6	112.9 (3)
C5—N3—C4	116.0 (3)	C9—C8—C6	104.9 (2)
C10—N4—C11	123.0 (2)	C9—C8—H8	127.5
C10—N4—H4	118.5	C6—C8—H8	127.5
C11—N4—H4	118.5	N2—C9—C8	106.3 (2)
C18—N5—C19	121.1 (2)	N2—C9—C10	122.9 (2)
C18—N5—H5	119.5	C8—C9—C10	130.7 (2)
C19—N5—H5	119.5	O1—C10—N4	124.3 (2)
C2—C1—C5	118.5 (3)	O1—C10—C9	121.2 (2)
C2—C1—Cl1	119.8 (3)	N4—C10—C9	114.5 (2)
C5—C1—Cl1	121.7 (2)	C16—C11—C12	120.8 (2)
C3—C2—C1	118.1 (3)	C16—C11—N4	119.4 (2)
C3—C2—H2	120.9	C12—C11—N4	119.8 (2)
C1—C2—H2	120.9	C13—C12—C11	118.8 (2)
C4—C3—C2	119.6 (3)	C13—C12—C18	120.3 (2)
C4—C3—H3	120.2	C11—C12—C18	120.9 (2)
C2—C3—H3	120.2	C14—C13—C12	119.7 (3)
N3—C4—C3	123.4 (3)	C14—C13—H13	120.1
N3—C4—H4A	118.3	C12—C13—H13	120.1
C3—C4—H4A	118.3	C13—C14—C15	121.4 (3)
N3—C5—C1	124.3 (3)	C13—C14—Cl2	118.8 (2)
N3—C5—N2	115.7 (3)	C15—C14—Cl2	119.8 (2)
C1—C5—N2	119.9 (2)	C16—C15—C14	120.5 (3)
N1—C6—C8	112.2 (3)	C16—C15—H15	119.7
N1—C6—C7	120.2 (3)	C14—C15—H15	119.7
C8—C6—C7	127.6 (3)	C15—C16—C11	118.6 (3)
F1'—C7—F2'	106.9 (8)	C15—C16—C17	119.3 (3)
F1'—C7—F3	131.5 (6)	C11—C16—C17	122.1 (3)
F2'—C7—F3	42.6 (7)	C16—C17—H17A	109.5
F1'—C7—F2	45.3 (7)	C16—C17—H17B	109.5
F2'—C7—F2	67.0 (8)	H17A—C17—H17B	109.5
F3—C7—F2	106.3 (4)	C16—C17—H17C	109.5
F1'—C7—F3'	105.2 (8)	H17A—C17—H17C	109.5
F2'—C7—F3'	104.1 (8)	H17B—C17—H17C	109.5
F3—C7—F3'	64.8 (8)	O2—C18—N5	120.9 (2)
F2—C7—F3'	134.7 (7)	O2—C18—C12	121.4 (2)
F1'—C7—F1	62.1 (7)	N5—C18—C12	117.7 (2)
F2'—C7—F1	132.1 (7)	N5—C19—H19A	109.5
F3—C7—F1	107.1 (4)	N5—C19—H19B	109.5
F2—C7—F1	104.8 (4)	H19A—C19—H19B	109.5
F3'—C7—F1	46.8 (8)	N5—C19—H19C	109.5
F1'—C7—C6	115.8 (6)	H19A—C19—H19C	109.5
F2'—C7—C6	113.3 (7)	H19B—C19—H19C	109.5
			
C6—N1—N2—C9	−0.5 (3)	N1—N2—C9—C10	177.2 (2)
C6—N1—N2—C5	−175.3 (2)	C5—N2—C9—C10	−8.7 (4)
C5—C1—C2—C3	−0.1 (4)	C6—C8—C9—N2	−0.2 (3)
Cl1—C1—C2—C3	−179.5 (3)	C6—C8—C9—C10	−176.6 (3)
C1—C2—C3—C4	1.5 (5)	C11—N4—C10—O1	−8.6 (4)
C5—N3—C4—C3	1.0 (4)	C11—N4—C10—C9	170.3 (2)
C2—C3—C4—N3	−2.0 (5)	N2—C9—C10—O1	−22.0 (4)
C4—N3—C5—C1	0.5 (4)	C8—C9—C10—O1	154.0 (3)
C4—N3—C5—N2	178.7 (2)	N2—C9—C10—N4	159.1 (2)
C2—C1—C5—N3	−1.0 (4)	C8—C9—C10—N4	−24.9 (4)
Cl1—C1—C5—N3	178.5 (2)	C10—N4—C11—C16	64.7 (3)
C2—C1—C5—N2	−179.1 (3)	C10—N4—C11—C12	−116.2 (3)
Cl1—C1—C5—N2	0.4 (4)	C16—C11—C12—C13	−3.9 (4)
N1—N2—C5—N3	−69.8 (3)	N4—C11—C12—C13	177.1 (2)
C9—N2—C5—N3	116.4 (3)	C16—C11—C12—C18	175.2 (2)
N1—N2—C5—C1	108.4 (3)	N4—C11—C12—C18	−3.8 (3)
C9—N2—C5—C1	−65.4 (4)	C11—C12—C13—C14	−0.3 (4)
N2—N1—C6—C8	0.4 (3)	C18—C12—C13—C14	−179.4 (3)
N2—N1—C6—C7	179.6 (3)	C12—C13—C14—C15	3.7 (5)
N1—C6—C7—F1'	11.8 (10)	C12—C13—C14—Cl2	−177.2 (2)
C8—C6—C7—F1'	−169.1 (10)	C13—C14—C15—C16	−2.8 (5)
N1—C6—C7—F2'	135.8 (10)	Cl2—C14—C15—C16	178.0 (3)
C8—C6—C7—F2'	−45.1 (11)	C14—C15—C16—C11	−1.4 (4)
N1—C6—C7—F3	−177.9 (4)	C14—C15—C16—C17	175.9 (3)
C8—C6—C7—F3	1.1 (6)	C12—C11—C16—C15	4.7 (4)
N1—C6—C7—F2	61.9 (5)	N4—C11—C16—C15	−176.2 (2)
C8—C6—C7—F2	−119.0 (5)	C12—C11—C16—C17	−172.5 (3)
N1—C6—C7—F3'	−107.7 (10)	N4—C11—C16—C17	6.6 (4)
C8—C6—C7—F3'	71.4 (11)	C19—N5—C18—O2	4.6 (4)
N1—C6—C7—F1	−57.1 (5)	C19—N5—C18—C12	−177.1 (3)
C8—C6—C7—F1	122.0 (4)	C13—C12—C18—O2	157.4 (3)
N1—C6—C8—C9	−0.2 (3)	C11—C12—C18—O2	−21.8 (4)
C7—C6—C8—C9	−179.3 (3)	C13—C12—C18—N5	−21.0 (4)
N1—N2—C9—C8	0.4 (3)	C11—C12—C18—N5	159.9 (2)
C5—N2—C9—C8	174.5 (3)		

### Hydrogen-bond geometry (Å, °)

**Table:**

*D*—H···*A*	*D*—H	H···*A*	*D*···*A*	*D*—H···*A*
N4—H4···O2	0.86	2.37	2.673 (3)	101
N4—H4···O2^i^	0.86	2.25	3.107 (3)	176
N5—H5···O1^ii^	0.86	2.16	2.915 (3)	146
C8—H8···O2^i^	0.93	2.50	3.168 (4)	129

Symmetry codes: (i) −*x*+1, −*y*+2, *z*; (ii) *x*+1/4, −*y*+7/4, *z*−1/4.
